# Vitamin D receptor gene is epigenetically altered and transcriptionally up-regulated in multiple sclerosis

**DOI:** 10.1371/journal.pone.0174726

**Published:** 2017-03-29

**Authors:** Teresa Ayuso, Patricia Aznar, Luis Soriano, Ander Olaskoaga, Miren Roldán, María Otano, Iratxe Ajuria, Gerardo Soriano, Francisco Lacruz, Maite Mendioroz

**Affiliations:** 1 Multiple Sclerosis Unit, Department of Neurology, Complejo Hospitalario de Navarra, Pamplona, Navarra, Spain; 2 Department of Neurology, Complejo Hospitalario de Navarra, Pamplona, Navarra, Spain; 3 Neuroepigenetics Laboratory, Navarrabiomed- Navarra Institute for Health Research (IdiSNA), Pamplona, Navarra, Spain; Roswell Park Cancer Institute, UNITED STATES

## Abstract

**Objective:**

Vitamin D deficiency has been linked to increased risk of multiple sclerosis (MS) and poor outcome. However, the specific role that vitamin D plays in MS still remains unknown. In order to identify potential mechanisms underlying vitamin D effects in MS, we profiled epigenetic changes in vitamin D receptor (*VDR)* gene to identify genomic regulatory elements relevant to MS pathogenesis.

**Methods:**

Human T cells derived from whole blood by negative selection were isolated in a set of 23 relapsing-remitting MS (RRMS) patients and 12 controls matched by age and gender. DNA methylation levels were assessed by bisulfite cloning sequencing in two regulatory elements of *VDR*. mRNA levels were measured by RT-qPCR to assess changes in VDR expression between patients and controls.

**Results:**

An alternative *VDR* promoter placed at exon 1c showed increased DNA methylation levels in RRMS patients (median 30.08%, interquartile range 19.2%) compared to controls (18.75%, 9.5%), p-value<0.05. Moreover, a 6.5-fold increase in *VDR* mRNA levels was found in RRMS patients compared to controls (p-value<0.001).

**Conclusions:**

An alternative promoter of the *VDR* gene shows altered DNA methylation levels in patients with multiple sclerosis, and it is associated with *VDR* mRNA upregulation. This *locus* may represent a candidate regulatory element in the genome relevant to MS pathogenesis.

## Introduction

Vitamin D deficiency has been linked to increased risk of multiple sclerosis (MS) and poor outcome [1–4]. In addition to calcium homeostasis, vitamin D shows immunomodulatory properties that are being widely studied. For instance, vitamin D regulates interleukin secretion in antigen presenting cells, ameliorates the immune control of mesenchymal stem cells and modulates Th17 immune response, the latter playing a critical role in autoimmune diseases [5–7]. Most of the biological effects of vitamin D are mediated by the vitamin D receptor (*VDR*), a highly conserved nuclear receptor that acts as a promiscuous transcription factor [8]. Nevertheless, the specific role that *VDR* gene plays in MS still remains unknown.

It has been lately suggested that epigenetic mechanisms would be involved in MS pathogenesis, since MS susceptibility is influenced by both genetic and environmental factors. DNA methylation at CpG sites (CpGs) in the genome is one of the most crucial epigenetic mechanisms and regulates gene expression and chromatin structure. Specifically, methylation of CpG islands located at gene promoters typically inhibits gene expression while CpG islands in promoters of actively transcribed genes are usually demethylated [9]. DNA methylation has become increasingly meaningful in many areas of research, including autoimmune and neurological diseases. Regarding MS, a number of recent studies have identified several pathological processes that are regulated by DNA methylation. For example, promoter regions of *FOXP3* (forkhead box P3) and *IL-17A (*interleukin 17A) genes are hypomethylated in T cells from untreated MS patients, suggesting that untreated MS patients may have an overrepresentation of circulating Tregs and Th17 cells [10]. Moreover, demyelinizating processes may be regulated by epigenetic mechanisms, as *PAD-2* (peptidil arginine deaminase type 2) promoter methylation is 25% reduced in MS patients, compared with controls. PAD-2 destabilizes the myelin basic protein (MBP), which becomes an antigen for T cells [11]. Most recently, genome-wide DNA methylation profiling of CD8+ T and CD4+ T cells has shown distinct DNA methylation profiles in relapsing-remitting (RRMS) compared to healthy controls [12]. Importantly, methylation patterns of cell-free plasma DNA has been proposed as potential biomarkers for MS [13]. For a more comprehensive review of DNA methylation changes in MS please see references 14–16 [14–16].

Here we profiled DNA methylation levels in two distinct regulatory elements of *VDR* gene in T cells isolated from RRMS patients and healthy controls matched by age and gender. Interestingly, we observed a significant increase of DNA methylation levels within an alternative promoter of the *VDR* gene placed at exon 1c. We also measured *VDR* mRNA expression levels in RRMS patients and controls to explore functional features of this differentially methylated locus in MS.

## Materials and methods

### Subjects and human T cells DNA samples

We recruited 23 relapsing remitting MS (RRMS) patients and 12 age and gender-matched healthy controls for this study. Patients were enrolled at the Multiple Sclerosis Unit from the *Complejo Hospitalario de Navarra* and controls were recruited among hospital workers or patient’s next of kin. All RRMS patients fulfilled the revised McDonald criteria [17] and none of the participants was under vitamin D supplementation when blood samples were collected.

For all 35 subjects T cells were isolated from whole blood by negative selection with RosetteSep^™^ Human T Cell Enrichment Cocktail kit (StemCell Technologies Inc, Vancouver, BC, Canada). Next, genomic DNA was extracted by standardized methods [18] and stored at -20°C until further use.

The study was approved by the Ethics Committee of the *Complejo Hospitalario de Navarra* for using human subjects and written informed consent was obtained from all subjects participating in the study.

### Vitamin D receptor methylation profiling

Genomic DNA (500 ng) was bisulfite converted using the EpiTect Bisulfite Kit (QIAGEN, Redwood City, CA, USA) according to the manufacturer’s instructions. Two promoter regions within *VDR* gene, the main promoter and an alternative promoter overlapping exon 1c, were amplified by bisulfite PCR. Genomic coordinates were obtained from GRCh37/Hg19 assembly. Primer pair sequences were designed by MethPrimer [19] and are listed in S1 Table. After purification, PCR products were cloned using the TopoTA Cloning System (Invitrogen, Carlsbad, CA, USA) and a minimum of 12 independent clones were sequenced for each examined subject and promoter region. Average methylation for a particular subject and promoter region was calculated by QUMA software [20].

### Vitamin D receptor mRNA expression analysis

Total RNA was isolated from peripheral blood leukocytes (PBL) using the TRIzol (Invitrogen, Carlsbad, CA, USA) standard protocol. Genomic DNA was removed with recombinant DNase (TURBO DNA-free^™^ Kit, Ambion, Inc., Austin, TX, USA). RNA integrity was checked by 1.25% agarose gel electrophoresis under denaturing conditions. Concentration and purity of RNA were both evaluated with NanoDrop spectrophotometer. Complementary DNA (cDNA) was reverse transcribed from 1000 ng total RNA with SuperScript^®^ III First-Strand Synthesis Reverse Transcriptase (Invitrogen, Carlsbad, CA, USA) after priming with oligo-d (T) and random primers. RT-qPCR reactions were performed in triplicate with Power SYBR Green PCR Master Mix (Invitrogen, Carlsbad, CA, USA) in a QuantStudio^™^ 12K Flex Real-Time PCR System (Applied Biosystems, Foster City, CA, USA). Sequences of primer pair were designed using Real Time PCR tool (IDT, Coralville, IA, USA) and are listed in S1 Table. Relative expression levels of *VDR* mRNA in a particular sample was calculated as previously described [21] and the geometric mean of *ACTB* and *TBP* genes were used to normalize expression values.

### 25(OH)D3 levels in serum

Vitamin D [25(OH)D3] levels were measured by chemiluminescence immunoassay with magnetic microparticles using the LIAISON^®^ 25 OH Vitamina D TOTAL Assay (DiaSorin, Inc., Stillwater, MN, USA) in serum samples of all 35 participants. Serum samples were collected at same time as blood for methylation measurement ± 1 month. Vitamin D deficiency was defined as serum 25(OH)D3 levels below 20ng/mL, based on the recommendations of the Institute of Medicine [22].

### Statistical data analysis

Statistical analysis was performed with SPSS 21.0 (IBM, Inc., USA). Differences with p-value < 0.05 were considered significant. Statistical significance for bisulfite and mRNA expression intergroup differences was assessed by Mann-Whitney U test. To assess differences between cases and controls for variables, Mann-Whitney U test and Chi-square were used. Spearman’s rank correlation coefficient was used to determine correlation between continuous variables. GraphPad Prism version 6.00 for Windows (GraphPad Software, La Jolla, CA, USA) was applied to draw graphs except for methylation figures that were obtained by QUMA software [20].

## Results

### Multiple sclerosis patients and controls

Characteristics of all the subjects included in the study are shown in Table 1. No differences were found between patients and controls regarding serum 25(OH)D3 levels, leukocyte or lymphocyte counts. All patients received a diagnosis of relapsing-remitting multiple sclerosis (RRMS). Median disease duration was 7 years, interquartile range (IQR) 12.2 years. Most patients (73.9%) had mild disease with an expanded disability scale score (EDSS) equal or below 3 at study entry. Only 4 patients met criteria for clinical activity and 2 for radiological activity [23] on magnetic resonance imaging performed within 1 year before study entry.

**Table 1:** Characteristics of subjects included in the study.

Variables	MS patients n = 23	Controls n = 12	p- value
Age, median (IQR)	43 (19)	38.5 (23.75)	0.595
Gender, female %	60.9	66.7	0.517
Smoking status, yes %	70	43.5	0.259
Leukocyte count (N*10^9/L), median (IQR)	6.8 (3.1)	6.05 (3.25)	0.327
Lymphocyte count (N*10^9/L), median (IQR)	1.90 (1.4)	2.30 (0.73)	0.294
25(OH)D3 levels ng/mL, median (IQR)	22 (10)	27.5 (19.25)	0.085
Evolution, median years (IQR)	7 (12.2)		
EDSS ≤ 3, %	82.6		
Activity, %	13		
Progression, %	11		
Steroids, %	4.3		
IFN ß, %	47.8		
Glatimer acetate, %	4.3		
Fingolimod, %	8.7		
Natalizumab, %	34.8		

MS: multiple sclerosis; n: number of subjetcs; IQR: interquartile range; EDSS: expanded disability scale score.

### *VDR* methylation levels are increased in T cells from RRMS sclerosis patients

**Fig 1:**
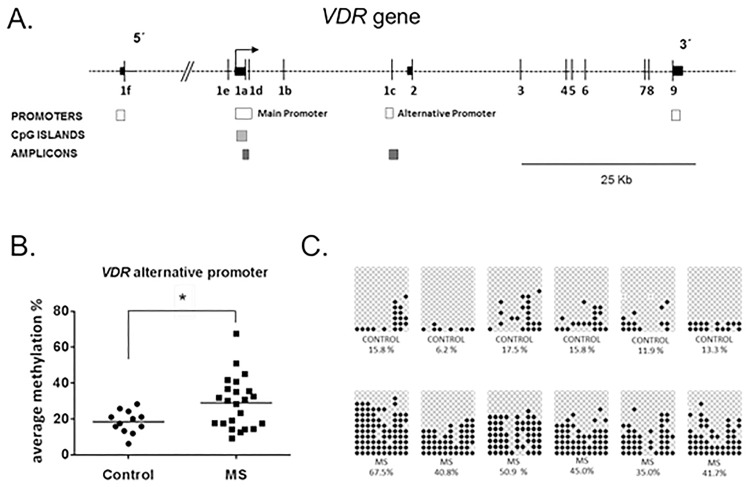
DNA methylation levels in *VDR* 1c promoter in T cells from RRMS patients and controls. (A) *VDR* map was adapted from Saccone et al., 2015 and shows relative position of promoter regions and the two amplicons surveyed by bisulfite sequencing cloning in our study: main and alternative *VDR* promoter regions. Vertical black lines represent exons, arrow represents transcription start site at the main promoter, white boxes show previously described promoter regions (Saccone et al., 2015), light grey boxes represent CpG islands and dark grey boxes symbolize the surveyed bisulfite sequencing amplicons. (B) The dot plot chart shows the significant increase in DNA methylation within the *VDR* alternative promoter in RRMS patients compared to controls. Horizontal lines represent median methylation values for each group. **(**C) Representative examples of bisulfite cloning sequencing for *VDR* alternative promoter are showed. In the upper line, several controls are presented while MS patients are depicted in the bottom line. Rectangles represent individual subjects. Black and white circles denote methylated and unmethylated cytosines, respectively. Each column symbolizes a unique CpG site in the examined amplicon and each line represents an individual DNA clone obtained from the bisulfite cloning sequencing. *p-value<0,05; ** p-value<0.005; MS = multiple sclerosis.

To begin to ask whether DNA methylation within vitamin D receptor (*VDR*) gene is altered in multiple sclerosis (MS), we examined the main promoter of *VDR* by bisulfite cloning sequencing. Main promoter region of *VDR* overlaps exon 1a and a CpG island located at the 5’ region of the gene (chr12:48298646–48299537; hg19 assembly). Most *VDR* transcripts are transcribed from that promoter. Primers were designed to amplify a 275 base-pair amplicon within the primary promoter region encompassing 23 CpG sites (Fig 1). DNA methylation percentage was measured at CpG site resolution and further averaged across all the CpG sites to calculate the average methylation level in the main promoter for each subject. Following that approach, we found that *VDR* main promoter was extensively demethylated [median (IQR), 2.6% (2.2%)], as corresponds to an actively expressed gene, and average methylation level was similar in T cells from RRMS patients and controls [2.85% (1.78%) vs. 1.1% (1%), p-value = 0.40].

We next decided to examine an alternative promoter located at non-coding exon 1c within the *VDR* gene body. We chose to measure DNA methylation at that particular promoter based on previous literature data [24]. It is a non-CpG island promoter that was reported to be regulated in a tissue-specific manner, being found to be hypomethylated in immune cells, stem cells and fetal pulmonary tissue [8]. A primer pair was designed to amplify a 345 base-pair fragment containing 10 CpG sites (Fig 1). *VDR* alternative promoter showed intermediate levels of DNA methylation [21.1% (23.5%)]. Interestingly, we found that average methylation level at *VDR* alternative promoter was significantly higher in T cells from RRMS patients compared to controls [30.08% (19.2%) vs. 18.75% (9.5%), p-value<0.05] (Fig 1, S2 Table), revealing a MS-related differentially methylated region located at a regulatory *locus* within the *VDR* gene.

We then analyzed the relationship between methylation levels at *VDR* alterative promoter and clinical variables listed in Table 1. There was no statistically significant relationship between DNA methylation levels and recorded clinical variables in our set of samples. Since the *VDR* alternative promoter could be bimodally distributed (Fig 1B) in RRMS patients, we tested differences in the phenotypic characteristics of patients by methylation levels (above versus below the median). No differences were found in clinical variables depending on the methylation level (S3 Table).

### *VDR* mRNA levels are upregulated in RRMS patients compared to healthy controls

**Fig 2:**
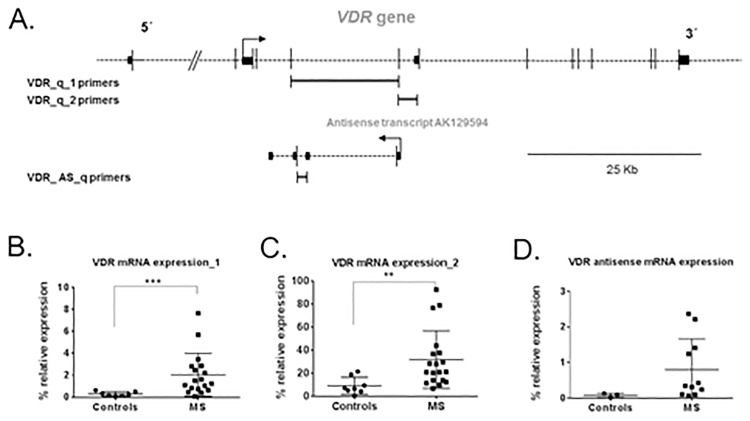
*VDR* mRNA expression is increased in multiple sclerosis patients. (A) The map shows fragments amplified by different set of primers for *VDR* gene. In the bottom line the map of *VDR* antisense AK129594 is shown. (B) The graph shows a significant 6.5-fold increase in *VDR* mRNA levels in MS patients compared to controls, when the first pair of primers (VDR_q_1 primers) was used. (C) A significant increase in *VDR* mRNA levels was also found when using the second pair of primers (VDR_q_2 primers) in the RT-qPCR reaction. (D) VDR antisense mRNA levels were increased in RRMS patients compared to controls but p-value did not reach statistical significance. Dots represent percentage of *VDR* expression relative to the geometric mean of *ACTB* and *TBP* housekeeping genes expression. Whiskers represent the standard error of the mean.** p-value<0.005; *** p-value<0.001.

To explore if *VDR* gene was also differentially expressed in RRMS patients compared to controls, we assessed *VDR* mRNA expression in peripheral blood leukocytes by real time quantitative PCR (RT-qPCR). To ensure reliable results, RT-qPCR reactions were performed in triplicate for each sample and repeated twice within independent cDNA sets. Moreover, two different set of primers (*VDR*-q1 and *VDR*-q2) (S1 Table, Fig 2A) were used to increase quality of RT-qPCR experiments. Interestingly, a 6.5-fold increase in *VDR* mRNA levels was found in RRMS patients compared to controls (p<0.001) (Fig 2B). When using a second pair of primers, a statistically significant increase in *VDR* mRNA expression was also found (p<0.005) (Fig 2C). We next measured mRNA expression levels of a natural antisense transcript (GenBank accession: AB307700) that originates from *VDR* alternative promoter overlapping exon 1c. This antisense transcript was expressed in our set of RRMS patients and controls. Notably, antisense *VDR* mRNA levels showed a trend to be increased in RRMS patients compared to controls (p = 0.060) but did not reach statistical significance in our set of samples (Fig 2D).

We further tested the relationship between *VDR* mRNA levels and clinical variables. A moderate inverse correlation was found between *VDR* mRNA expression and lymphocyte count (rSpearman = -0.502, p<0.05) (S1 Fig). Interestingly, a significant inverse correlation was also found between *VDR* mRNA expression and serum 25(OH)D3 levels (rSpearman = -0.412, p<0.05) (S1 Fig). No other association was found between *VDR* mRNA levels and smoking status, disease severity or treatment.

## Discussion

Here we report epigenetic changes in a regulatory region of *VDR* gene in RRMS patients. *VDR* gene is essential in mediating the pleiotropic biological effects of vitamin D. An important finding of this study is that a regulatory element within *VDR*, previously found to be hypomethylated in immune cells, is differentially methylated in T cells from RRMS patients compared to healthy controls.

Epigenetic regulation of the human *VDR* gene is a very highly complex process and represents a paradigm of gene-environment interaction through epigenetics [8]. Expression of *VDR* gene depends on four different promoters which give rise to 12–14 alternatively spliced transcripts [8]. One of the alternative promoters is placed within the gene body overlapping non-coding exon 1c (Fig 1) [24] and shows little conservation between species. Therefore, transcription originating from this region may occur in a tissue-specific manner or under pathological conditions [8,25]. Indeed, the *VDR* alternative promoter at exon 1c was found to be unmethylated in immune cells, according to Roadmap Epigenomics and ENCODES project data [8], suggesting that its regulation may play a role in immune function. Supporting this idea, we found intermediate levels of DNA methylation in T cells for the *VDR* alternative promoter at exon 1c in our set of samples. What is more interesting, a significant increase in DNA methylation within this alternative promoter was shown in T cells from RRMS patients compared to controls in our study (Fig 1), suggesting that epigenetic regulation of *VDR* alternative promoter at exon 1c may be altered in MS patients. The role that this alternative promoter plays in immune function and whether this alteration is specific to MS or shared by other autoimmune conditions will need further investigation.

We also found a significant upregulation of *VDR* mRNA levels in RRMS patients compared to healthy controls (Fig 2) in PBLs, as an indirect approach to VDR expression in the T-cell population. This result is in contrast with a previous study performed on CD4+ T cells isolated from RRMS patients and healthy controls [26], where authors did not found significant differences in *VDR* mRNA levels between both groups. There are a number of reasons that may account for such differences. Total RNA was isolated from different cell populations, being peripheral blood leucocytes the source of RNA in our study, whereas CD4+ T cells were used in the referred study. As stated in the paper, RNA was extracted after 3 days of culture, which also may have introduced some changes in *VDR* mRNA levels. In addition, we utilized the geometric mean of two housekeeping genes, *ACTB* and *TBP*, which are different from the housekeeping gene used in the reported paper that was *GAPDH*. On the contrary, our finding is in concordance with a previous report that showed upregulation of *VDR* mRNA in brain tissue from MS patients. *VDR* mRNA levels were higher in active MS lesions compared to MS normal-appearing white matter. Interestingly, authors also found an increase in *VDR* mRNA levels in MS normal-appearing white matter compared to control white matter [27]. Furthermore, according to our study, *VDR* mRNA expression has been found to be increased in T cells in other autoimmune diseases, such as systemic lupus erythematosus [28] (S2 Fig).

To extend the assessment of *VDR* mRNA expression, we also measured mRNA levels of a particular type of transcript (Fig 2). According to GenBank data obtained through the UCSC Genome Browser, there is a 4-exon transcript (GenBank accession: AK129594) originating from *VDR* alternative promoter at exon 1c, which is known to be expressed in cancerous tissues and germinal center B cells, among other tissues [8]. Remarkably, this transcript is a natural antisense transcript (NAT) and as such, it might influence *VDR* mRNA expression. NATs are important modulators of gene expression and they may be part of self-regulatory circuits that allow genes to regulate their own expression [29]. We hypothesized that *VDR* antisense transcript (GenBank accession: AK129594) would have a silencing function and its mRNA expression would be reduced in RRMS patients, then allowing higher expression of main transcripts of *VDR* gene. Nevertheless, in our set of samples, a trend was found towards *VDR* mRNA expression being higher in RRMS patients (Fig 2D), which contradicts the previous hypothesis.

In summary, this study provides evidence that an alternative promoter at exon 1c of the *VDR* gene shows aberrant DNA methylation in T cells from RRMS patients. VDR mRNA expression was also shown to be upregulated in PBLs from RRMS patients compared to healthy controls. On the whole, these results suggest that epigenetic mechanisms may underlie the role that vitamin D plays in MS. Further functional and mechanistic studies will help to better understand the biological significance and importance of the alternative *VDR* promoter in the regulation of *VDR* in immune cells. In a translational level, methylation levels of *VDR* alternative promoter may be worthy to explore as a candidate biomarker in MS patients.

## Supporting information

S1 Fig Correlation between VDR mRNA expression and biological variables. The dot-plot charts A and B show an inverse moderate correlation of*VDR* mRNA expression with lymphocyte count (N*10^9/L) (A) and serum 25(OH)D3 (calcidiol) levels, ng/mL (B).
(PDF)

S2 Fig Gene Expression Omnibus (GEO) data analysis for VDR mRNA expression levels in Systemic Lupus Erythematosus (SLE). The bar chart represents VDR mRNA expression levels obtained from the GEO database for the Affymetrix Human Genome U133 Plus 2.0 Array performed on T cells from SLE patients and controls. *p-value<0.05.
(PDF)

S1 Table Bisulfite and RT-qPCR PCR primers. (PDF)

S2 Table Methylation levels for each CpG site in control and MS groups within VDR alternative promoter amplicon. (PDF)

S3 Table Characteristics of patients depending on methylation levels of the VDR 1c alterative promoter (below versus above the median). (PDF)
