# Supplementary material for: Vitamin D receptor gene is epigenetically altered and transcriptionally up-regulated in multiple sclerosis
Source: PLoS One. 2017 Mar 29;12(3):e0174726. doi: 10.1371/journal.pone.0174726 (PMC5371344; doi:10.1371/journal.pone.0174726)
Supplement: S1 Fig — The dot-plot charts A and B show an inverse moderate correlation ofVDR mRNA expression with lymphocyte count (N*10^9/L) (A) and serum 25(OH)D3 (calcidiol) levels, ng/mL (B). (PDF) [file pone.0174726.s001.pdf]

S1 Fig. Correlation between VDR mRNA expression and biological variables.

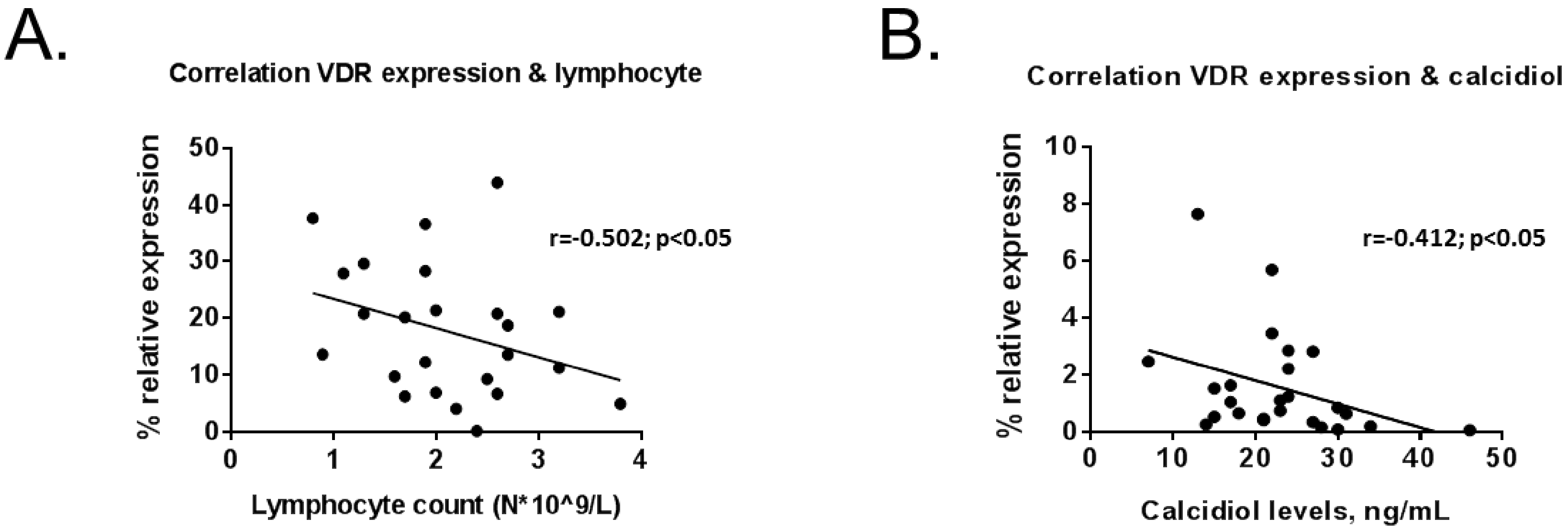

The dot-plot charts A and B show an inverse moderate correlation of*VDR* mRNA expression with lymphocyte count (N*10^9/L) (A) and serum vitamin D (calcidiol) levels, ng/mL (B).
