# Supplementary material for: Vitamin D receptor gene is epigenetically altered and transcriptionally up-regulated in multiple sclerosis
Source: PLoS One. 2017 Mar 29;12(3):e0174726. doi: 10.1371/journal.pone.0174726 (PMC5371344; doi:10.1371/journal.pone.0174726)
Supplement: S2 Fig — The bar chart represents VDR mRNA expression levels obtained from the GEO database for the Affymetrix Human Genome U133 Plus 2.0 Array performed on T cells from SLE patients and controls. *p-value<0.05. (PDF) [file pone.0174726.s002.pdf]

S2 Fig. Gene Expression Omnibus (GEO) data analysis for *VDR* mRNA expression levels in Systemic Lupus Erythematosus (SLE).

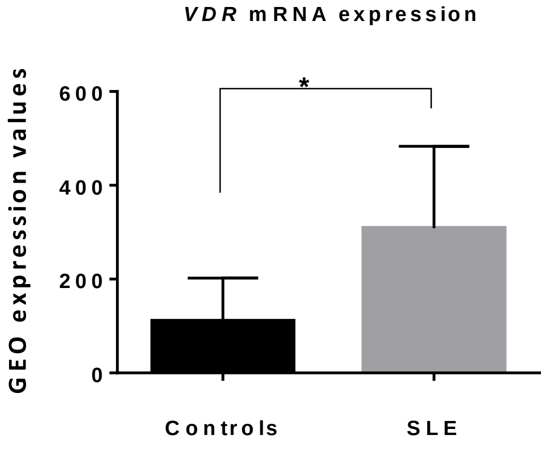

The bar chart represents *VDR* mRNA expression levels obtained from the GEO database for the Affymetrix Human Genome U133 Plus 2.0 Array performed on T cells from SLE patients and controls. *p-value<0.05.
