# Supplementary material for: Vitamin D receptor gene is epigenetically altered and transcriptionally up-regulated in multiple sclerosis
Source: PLoS One. 2017 Mar 29;12(3):e0174726. doi: 10.1371/journal.pone.0174726 (PMC5371344; doi:10.1371/journal.pone.0174726)
Supplement: S1 Table — (PDF) [file pone.0174726.s003.pdf]

S1 Table. Bisulfite and RT-qPCR PCR primers

Identification	PCR Purpose	Amplicon size	Tm	Forward Primer	Tm2	Reverse Primer	CpGs in amplicon
VDR_MP_bis	Bisulfite PCR	275 bp	59.29	GTTGGGTTGTTTTTGTTTG TTAAA	58.8	TCAAACCTCAATACCCCTTA ATATC	23
VDR_AP1c_bis	Bisulfite PCR	345 bp	57.39	TTTAAAGGTTTTTGAAATG AAGAAG	59.44	CTCCCTCCTAACACCCTAAA CTATAA	10
VDR_q_1	qPCR	128 bp	62.3	GCTGGACGGAGAAATGGAC	62	ACAGACACTTCAGACCCAAAG	NA
VDR_q_2	qPCR	135 bp	62.2	TTTGGGTCTGAAGTGTCTG TG	62.1	GTTCCGGTCAAAGTCTCCAG	NA
VDR_AS_q	qPCR	150 bp	61.7	CCATTCACCCTCGTACCAC	62.2	GTTCTCCATCCTTTTCAACA CTTG	NA

The table shows the primer pairs used in the study. Amplified transcripts are identified by RefSeq Accession or GeneBank accesion number.

qPCR: quantitative PCR; bp: base pair; Tm: Melting Temperature; NA: not applicable
