# Supplementary material for: Vitamin D receptor gene is epigenetically altered and transcriptionally up-regulated in multiple sclerosis
Source: PLoS One. 2017 Mar 29;12(3):e0174726. doi: 10.1371/journal.pone.0174726 (PMC5371344; doi:10.1371/journal.pone.0174726)
Supplement: S2 Table — (PDF) [file pone.0174726.s004.pdf]

S2 Table. Methylation levels for each CpG site in control and MS groups within VDR alternative promoter amplicon

CpG position	46	51	58	87	172	195	226	257	263	298	Median	IQR
MR control group, average	0.22	0.17	0.25	0.13	0	0.17	0.04	0.37	0.32	0.19	0.19	0.09
MR multiple sclerosis group, average	0.25	0.33	0.36	0.25	0.08	0.25	0.17	0.42	0.45	0.25	0.3	0.19
Gain in methylation, %	3	16	11	12	8	8	13	5	13	6	11	-
p-value	0.079	0.034	0.115	0.041	0.221	0.045	0.062	0.263	0.031	0.057	0.034	-

Methylation ratio was calculated as the ratio of methylated CpGs over the total number of CpGs assessed. Gain in methylation was calculated as the subtraction of percentage of methylation between the AD group and control group. Mann-Whitney U-test was used to assess differences between control and AD groups. CpG position denotes the position of the dinucleotide CpG within the amplicon. MR: methylation ratio; MS: Multiple Sclerosis; IQR: interquartile range
