# Supplementary material for: Vitamin D receptor gene is epigenetically altered and transcriptionally up-regulated in multiple sclerosis
Source: PLoS One. 2017 Mar 29;12(3):e0174726. doi: 10.1371/journal.pone.0174726 (PMC5371344; doi:10.1371/journal.pone.0174726)
Supplement: S3 Table — (PDF) [file pone.0174726.s005.pdf]

S3 Table. Characteristics of patients depending on methylation levels of the *VDR* 1c alterative promoter (below versus above the median)

Variables	Methylation level < 30.08% n=12	Methylation level > 30.08% n=11	p- value
Age, median (IQR)	45 (19)	33 (21)	0.151
Gender, female %	58.3	63.6	0.795
Smoking status, yes %	41.7	45.5	0.855
Leukocyte count (N*10^9/L), median (IQR)	6.95 (2.55)	6.4 (4.3)	0.786
Lymphocyte count (N*10^9/L), median (IQR)	2.25 (1.9)	1.6 (1.3)	0.449
25(OH)D3 levels ng/mL, median (IQR)	19.5 (7.25)	24 (8)	0.079
Evolution, median years (IQR)	11.5 (14.5)	7 (6)	0.651
EDSS ≤ 3, %	75	90.9	0.315
Activity, %	25	9.1	0.315
Progression, %	8.3	0	0.328
Steroids, %	0	9.1	0.286
IFN ß, %	33.3	63.6	0.146
Glatimer acetate, %	8.3	0	0.328
Fingolimod, %	8.3	9.1	0.949
Natalizumab, %	50	18.2	0.110
